# Effect of an α-calcium sulfate hemihydrate/treated dentin matrix composite to regenerate bone in critically sized SD rat calvarial defects

**DOI:** 10.3389/fbioe.2024.1468296

**Published:** 2024-12-20

**Authors:** Mengmeng Wang, Bingyan Li, Wenshuai Li, Zelong Hu, Haojie Fu, Rui Li

**Affiliations:** ^1^ Department of Stomatology, Jiading District Central Hospital Affiliated Shanghai University of Medicine and Health Sciences, Shanghai, China; ^2^ Department of Stomatology, The First Affiliated Hospital of Zhengzhou University, Zhengzhou, China

**Keywords:** treated dentin matrix, bone, stem cell, tissue engineering, bone regeneration

## Abstract

**Introduction:**

α-Calcium sulfate hemihydrate (α-CSH) is a widely used artificial bone graft material, but it suffers from rapid deterioration and limited osteoinductivity. This study aims to develop composite cements by combining treated dentin matrix (TDM) with α-CSH to enhance osteogenic properties for the healing of bone deformities.

**Methods:**

The composite cements were prepared by mixing treated dentin matrix (TDM) with α-calcium sulfate hemihydrate (α-CSH) and characterized for their mechanical, morphological, and chemical properties using a universal mechanical testing machine, scanning electron microscopy (SEM), X-ray diffraction (XRD), and Fourier-transform infrared (FTIR) spectroscopy. The biological performance was assessed by measuring osteoblast proliferation using the CCK-8 test and evaluating new bone formation in a calvarial bone defect model in rats.

**Results:**

The compressive strength of the TDM/α-CSH cements decreased with increasing TDM mass ratio, while SEM analysis revealed a distinct three-dimensional porous network structure and increased surface roughness upon thorough mixing. XRD and FTIR studies confirmed the physical mixture of the two materials without phase changes. The TDM/α-CSH composites significantly stimulated osteoblast proliferation, which was dependent on the TDM content, and demonstrated superior enhancement in new bone formation as confirmed by X-ray examination and micro-CT analysis.

**Discussion:**

The findings suggest that TDM/α-CSH composite cements have promising potential as an alternative for repairing bone defects due to their improved mechanical properties, osteoblast proliferation, and enhanced new bone formation *in vivo*.

**Conclusion:**

TDM/α-CSH composite cements show potential as a novel bone graft material, offering advantages in terms of mechanical strength, osteoconductivity, and osteoinductivity, making them a viable option for bone repair applications.

## Graphical Abstract

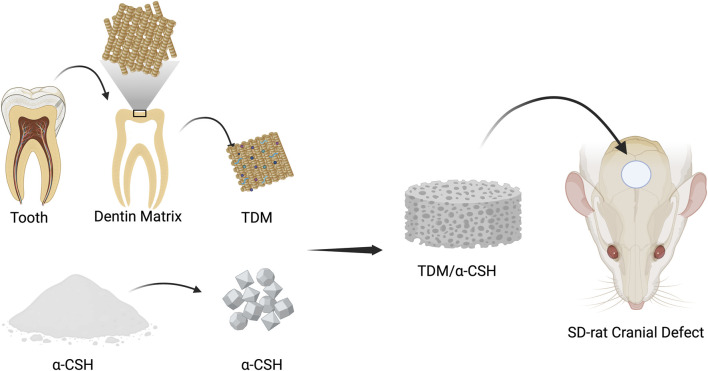

## 1 Introduction

Due to tumors, inflammation, trauma, congenital deformity, spinal fusion, and other reasons, bone destruction and defect of different degrees are very common in clinics. Autogenous and allogeneic bone transplantations are the most commonly used and effective means of repairing bone defects. However, autogenous bone transplantation has some shortcomings, such as fewer bone sources, additional trauma to patients, and bone resorption of autogenous bone in the recipient area after operation (Mao et al., 2009; Chen et al., 2012; Ewers et al., 2004; Ebraheim et al., 2001). The osteoinductive capacity of allogeneic and xenogeneic bone is relatively limited, and concerns regarding immune rejection, as well as the potential transmission of HIV, hepatitis B, and other infectious diseases, significantly restrict their clinical application (Nauth et al., 2011; Chen and Jin, 2010). α-Calcium sulfate hemihydrate (α-CSH) has been used in clinical bone defect repair for more than 100 years as a substitute material for artificial bone transplantation because of its low cost, uniform structure, and easy molding; in addition, a large number of experimental studies have confirmed that it has excellent osteoconductive, biocompatible, and biodegradable properties (Turner et al., 2003; Strocchi et al., 2002; Kim et al., 2011; Thomas and Puleo, 2009). Effective bone repair requires a balance between material resorption and new bone formation. The primary drawback of α-CSH *in vivo* is its rapid degradation, which outpaces the rate of new bone formation. Typically, α-CSH is completely degraded within 4–8 weeks, creating a “vacuum zone” where new bone has not yet filled the defect, leading to fibrous tissue ingrowth and immature bone formation (Gao et al., 2007; Ball et al., 2014; Beuerlein and Mckee, 2010; Orsini et al., 2004). Additionally, α-CSH lacks osteoinductive properties. Therefore, to address the challenges of bone tissue defect repair, the development of bone graft substitutes based on α-CSH with more favorable characteristics has garnered increasing attention.

Treated partially demineralized dentin has received considerable attention recently. Approximately 65% of the hard-tissue dentin is composed of hydroxyapatite (HA). Furthermore, 95% of the organic components are type I collagen (Col-I), and studies have shown that type I collagen promotes the formation and calcification of new bone (Prasad et al., 2010). The composition of this substance is very similar to that of normal bone tissue. A large number of studies have found that HA can obtain a satisfactory bone repair effect without causing immune rejection, inflammation, and foreign-body reaction, which has good biocompatibility after implantation *in vivo* (Shigeru et al., 1998; Frame et al., 1987; Matthew et al., 2002; Munro et al., 2007). In addition, there are some non-collagen proteins related to osteogenesis, such as osteopontin, osteocalcin, bone morphogenetic protein BMP-2, and dentin matrix protein DMP1. In the 1960s, Yeomans and Urist (1967) used partially demineralized dentin as a substitute for bone transplantation to study its osteogenic properties, and the results showed that the osteogenic induction ability of partially demineralized dentin was significantly higher than that of well-mineralized dentin without demineralization. Numerous experimental studies have demonstrated that partially demineralized dentin exhibits superior osteogenic and bone conductive properties to HA when used alone for the repair of bone tissue defects. However, one of the major drawbacks of partially demineralized dentin is its hardly moldable properties. As a substitute material for bone transplantation, its safety has also been unanimously recognized by many scholars (Dioguardi et al., 2015; Kim et al., 2007).

Based on the previous analysis, this study prepared a treated dentin matrix (TDM) through gradient demineralization and combined it with α-CSH at different mass ratios to synthesize an α-CSH/TDM composite. This approach aims to overcome the limitations of single-component materials and better meet the requirements for clinical bone tissue defect repair.

## 2 Materials and methods

### 2.1 Preparation and characterization of the TDM/α-CSH composite

#### 2.1.1 Fabrication of the TDM

##### 2.1.1.1 Preparation of the TDM

Healthy and intact teeth, extracted due to clinical orthodontic treatment, were collected from the outpatient department of stomatology at the First Affiliated Hospital of Zhengzhou University. The cementum and a portion of the dentin from the crown were removed, and the entire periodontal tissue was carefully scraped off using a periodontal curette. Additionally, some outer dentin and cementum were ground along the teeth profile. The predentin and pulp tissues were thoroughly cleaned using root canal instruments.

The prepared dentin matrix was soaked in phosphate-buffered saline (PBS) for 30 min, followed by multiple rounds of ultrasonic cleaning (3–8 cycles), each lasting 20 min. The dentin matrix was then cryopreserved in tubes containing a preservation solution and stored in liquid nitrogen.

Subsequently, the matrix was freeze-dried using a vacuum freeze dryer (FreeZone^®^ Triad™ 2.5 L, Labconco, United States) for 8 h. The dried dentin was mechanically ground in a high-throughput tissue grinder (DHS Dinghaoyuan Technology Company, PRC) at 1,200 rpm until the particles passed smoothly through a 50-mesh sieve. These particles were then subjected to gradient demineralization with 17% ethylene diamine tetra-acetic acid (EDTA; Sigma, United States) for 10 min, 10% EDTA for 10 min, and 5% EDTA for 20 min. After each demineralization step, the particles were rinsed in deionized water using an ultrasonic cleaner. The resulting TDM was freeze-dried again, weighed, repackaged, sterilized by Co-60 irradiation, and stored at −80°C for subsequent use.

##### 2.1.1.2 Preparation of TDM/α-CSH composite bone graft materials

The TDM was mixed with α-CSH (Sigma, United States) at mass ratios of 0%, 10%, 30%, and 50%. The mixtures were combined with deionized water at a liquid-to-solid ratio of 0.4 and stirred using a magnetic stirrer (Thermo Fisher Scientific, United States) for approximately 60 s until a homogeneous paste was obtained.

##### 2.1.1.3 Mechanical testing of TDM/α-CSH composites

The TDM/α-CSH composite was transferred into a Teflon mold with cylindrical cavities (20 mm in height and 8 mm in diameter). Both ends of the mold were pressed between two metal plates until the composite hardened. After 2 h, the samples were removed from the mold and incubated at 37°C for 10 days. Mechanical testing was performed using a universal testing machine (WDW-5C, Shanghai Hualong Co. Ltd., China) with a loading rate of 0.5 mm/min. Deflection and load were recorded until failure, with five samples tested per group. The compressive strength and elastic modulus were calculated using a computer.

##### 2.1.1.4 Scanning electron microscopy analysis

A 200-μL suspension of α-CSH or TDM/α-CSH in absolute ethanol was applied to a copper stub and allowed to air-dry at room temperature. Once dry, the samples were coated with gold using a vacuum sputter coater. The micromorphology of the crystal surfaces was then observed using a scanning electron microscope (SEM; SU8010, Hitachi, Japan).

##### 2.1.1.5 X-ray diffraction characterization

The crystalline phases of α-CSH and TDM/α-CSH composites at different mass ratios were analyzed using X-ray diffraction (XRD; PANalytical X'Pert PRO, Netherlands) under a current of 40 mA and a voltage of 40 kV. Scanning was performed at 4°/min over a 2θ range of 10°–70°, with a step size of 0.02°.

##### 2.1.1.6 Fourier transform infrared spectroscopy

Samples of α-CSH and TDM/α-CSH composites, ground to a particle size smaller than 200 mesh, were mixed with KBr at a 1:100 ratio. The mixture was then compressed into pellets and analyzed using Fourier-transform infrared (FTIR) spectroscopy (VERTEX70, Bruker, Germany) in the range of 4,000 cm⁻^1^–375 cm⁻^1^ with a resolution of 4 cm⁻^1^.

### 2.2 Extraction and cultivation of primary osteoblasts

8-week-old Sprague–Dawley (SD) suckling rats of SPF grade were obtained from the Henan Experimental Animal Center. After euthanasia via cervical dislocation, the rats were soaked in 75% ethanol for approximately 30 min. The calvarial bones were then extracted and cut into 1 × 1 × 1 cm³ fragments, which were transferred to a T25 culture flask containing 5 mL of 0.25% trypsin (Gibco, United States). Subsequently, the digestion process was continued using 0.1% type I collagenase (Sigma, United States). The treated bone fragments were then transferred to Dulbecco’s modified Eagle’s medium (DMEM; HyClone, United States) supplemented with 15% fetal bovine serum (FBS; HyClone, United States) and 1% penicillin–streptomycin (100 U/mL penicillin and 100 mg/mL streptomycin). After incubation at 37°C in a humidified atmosphere with 5% CO₂ for approximately 4 h, the culture flask was gently returned to its upright position once the bone fragments had adhered to the flask wall. A fresh culture medium was added, and the cells were further incubated.

### 2.3 Alizarin Red S staining of osteoblasts

The purified and passaged osteoblasts from a P3 cell suspension were seeded into 6-well plates at a density of 5 × 10⁴ cells per well. The cells were cultured in a humidified incubator at 37°C with 5% CO₂ for 25 days without further subculturing, with the growth medium being replaced every 2–3 days. After the incubation period, the cells were stained with 0.2% Alizarin Red S (Solarbio, PRC) and observed under a fluorescence microscope (NIKON DI, Nikon, Japan).

### 2.4 Alkaline phosphatase staining of osteoblasts

The purified and passaged osteoblasts from the P3 cell suspension were seeded into 6-well plates at a density of 5 × 10⁴ cells per well. The cells were incubated in a humidified incubator at 37°C with 5% CO₂ for 5 days, with the culture medium being replaced every 2–3 days. Once the osteoblasts reached approximately 80% confluence, they were stained with alkaline phosphatase (Solarbio, PRC) and examined under a fluorescence microscope (NIKON DI, Nikon, Japan).

### 2.5 Drawing of osteoblast cell growth curve using the CCK-8 assay

In accordance with ISO 10993 guidelines, each set of materials sterilized via Co-60 irradiation was submerged in DMEM containing 10% FBS and 1% penicillin–streptomycin at a concentration of 0.2 g/mL. The materials were incubated for 10 days at 37°C in a 5% CO₂ atmosphere. The supernatant was then collected, filtered through a 0.22 μm filter, and stored at 4°C for future experiments. The purified and passaged osteoblasts from the P3 cell suspension were seeded into 96-well plates at a density of 2 × 10³ cells per well. Each experimental group included one empty well and five additional wells. The empty well, containing DMEM with 10% FBS and 1% penicillin–streptomycin, served as the control group. After 24 h of incubation at 37°C with 5% CO₂, the medium was removed once the osteoblasts adhered to the well surface, and each well was washed twice with PBS. Subsequently, 100 μL of the material extract from each sample group was added to the wells, and the cells were cultured for 1–8 days. Before conducting the assay, the supernatant was removed, and 100 μL of DMEM, along with 10 μL of CCK-8 reagent, was added to each well. The absorbance was measured at 450 nm using a microplate reader (Multiskan FC, Thermo Fisher Scientific, United States) after an additional 3 h incubation under 5% CO₂ at 37°C.

### 2.6 Transplantation of the SD rat critical calvarial bone defect model

All animal procedures were approved by the Ethics Committee of the First Affiliated Hospital of Zhengzhou University, People’s Republic of China. Male SD rats, 8 weeks old and weighing approximately 250 g ± 30 g, were obtained from the Henan Experimental Animal Centre. After acclimatization, the rats were anesthetized via intraperitoneal injection of 3% pentobarbital at a dose of 50 mg/kg. A V-shaped incision was made on the scalp of the parietal region, exposing the skull by separating the soft tissues. A 5-mm-diameter implant trephine was used to create a full-thickness critical-sized calvarial defect (5 mm) at the central part of the calvarium, above the sagittal suture and anterior to the lambdoid suture. The trephine was continuously cooled with 0.9% saline throughout the procedure. Each material set was mixed with sterilized deionized water at a liquid-to-solid ratio of 0:4 (Yang et al., 2017a) and then solidified in a Teflon mold to form sample blocks of 5 mm in diameter and 1 mm in thickness. A total of 40 rats were randomly divided into 5 groups, with the calvarial defects filled with α-CSH, 10% TDM/α-CSH, 30% TDM/α-CSH, and 50% TDM/α-CSH sample blocks, while the control group remained unfilled. The incisions were then sutured in layers, and postoperative care was administered. Each rat received a daily intramuscular injection of 800,000 units of penicillin sodium into the thigh for 3 consecutive days.

### 2.7 X-ray examination

Twelve weeks post-operation, the experimental animals in each group were euthanized, and the calvarial defects, along with the surrounding tissue, were carefully dissected. The harvested specimens were then fixed in 10% formaldehyde solution for further analysis. X-ray imaging was conducted, and the area of new bone formation, as well as the percentage of defect coverage, was calculated using Image-Pro Plus 6.0 software.

### 2.8 Micro-computed tomography measurement

Micro-computed tomography (micro-CT) measurements were performed using a SkyScan 1176 μ-CT system (Bruker Co. Ltd., Germany) with a source voltage of 58 kV and a source current of 431 μA. The image pixel size was set to 9 μm, with an exposure time of 1,000 ms. Three-dimensional (3D) images were reconstructed using NRecon software.

### 2.9 Immunohistochemical analysis

Immunohistochemistry was performed using the following antibodies: rabbit anti-OPN (Abcam, Inc., United Kingdom), rabbit anti-Col-I (Abcam, Inc., United Kingdom), rabbit anti-Col-II (Abcam, Inc., United Kingdom), and goat anti-rabbit secondary antibodies. Sample sections were prepared through a series of steps including decalcification with EDTA for 28 days, followed by dehydration in an ascending ethanol series. After paraffin embedding, the samples were sectioned into 5-μm-thick slices. For visualization, the sections were stained with NovaRED and hematoxylin. Finally, the specimens were examined using a fluorescence microscope (Olympus U-RFLT50, Japan).

### 2.10 Statistical analysis

Data processing and analysis were performed using SPSS 20.0 software. Measurement data were expressed as the mean ± standard deviation. Statistical comparisons between groups were conducted using the *t*-test. A *p*-value of less than 0.05 was considered statistically significant.

## 3 Results

### 3.1 Characterization of the TDM/α-CSH composite

**FIGURE 1:**
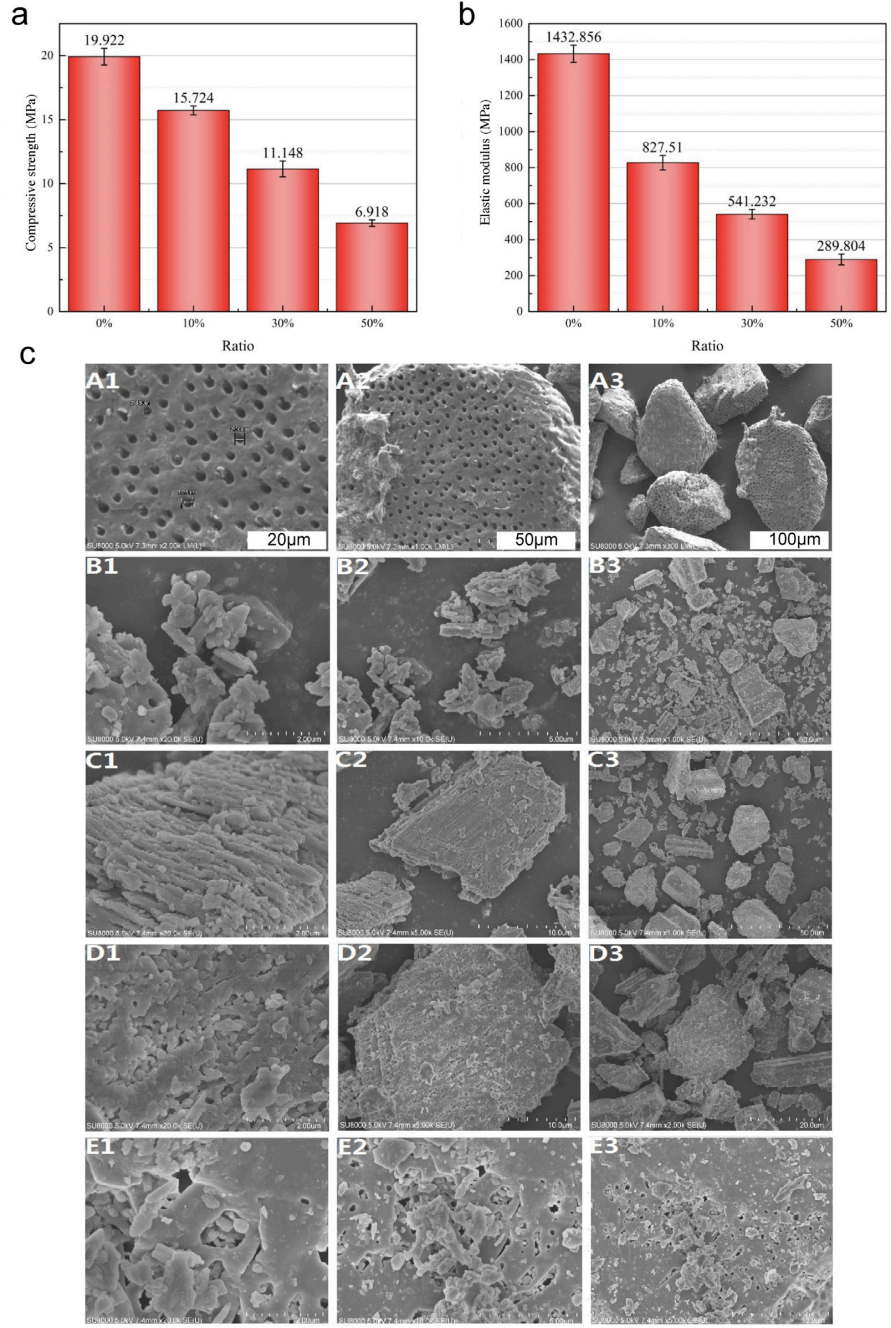
Characterization of the TDM/α-CSH composite included the evaluation of average compressive strength **(A)** and elastic modulus **(B)** for α-CSH and the 10% TDM/α-CSH, 30% TDM/α-CSH, and 50% TDM/α-CSH composite cements. Statistical differences were observed between each pair of groups (*p* < 0.05). **(C)** SEM images of the TDM (A1–A3), α-CSH (B1–B3), 10% TDM/α-CSH (C1–C3), 30% TDM/α-CSH (D1–D3), and 50% TDM/α-CSH (E1–E3) materials at various magnifications.

The average compressive strength and elastic modulus of the TDM/α-CSH cements, as determined by the universal mechanical testing equipment, decreased as the amount of TDM incorporation increased (see Figures 1A, B). Furthermore, as the mass ratio of TDM incorporation increased, the maximal mechanical load value of the composite material gradually decreased at the point of critical failure. The mean compressive strengths of 0% TDM/α-CSH, 10% TDM/α-CSH, 30% TDM/α-CSH, and 50% TDM/α-CSH were calculated to be 19.92 ± 0.64 MPa, 15.72 ± 0.34 MPa, 11.14 ± 0.61 MPa, and 6.91 ± 0.24 MPa, respectively. The average elastic moduli were 1,432.85 ± 47.69 MPa, 827.51 ± 40.13 MPa, 541.23 ± 26.44 MPa, and 289.80 ± 29.92 MPa, respectively. Additionally, statistical differences were observed between each pair of categories (*p* < 0.05).

**FIGURE 2:**
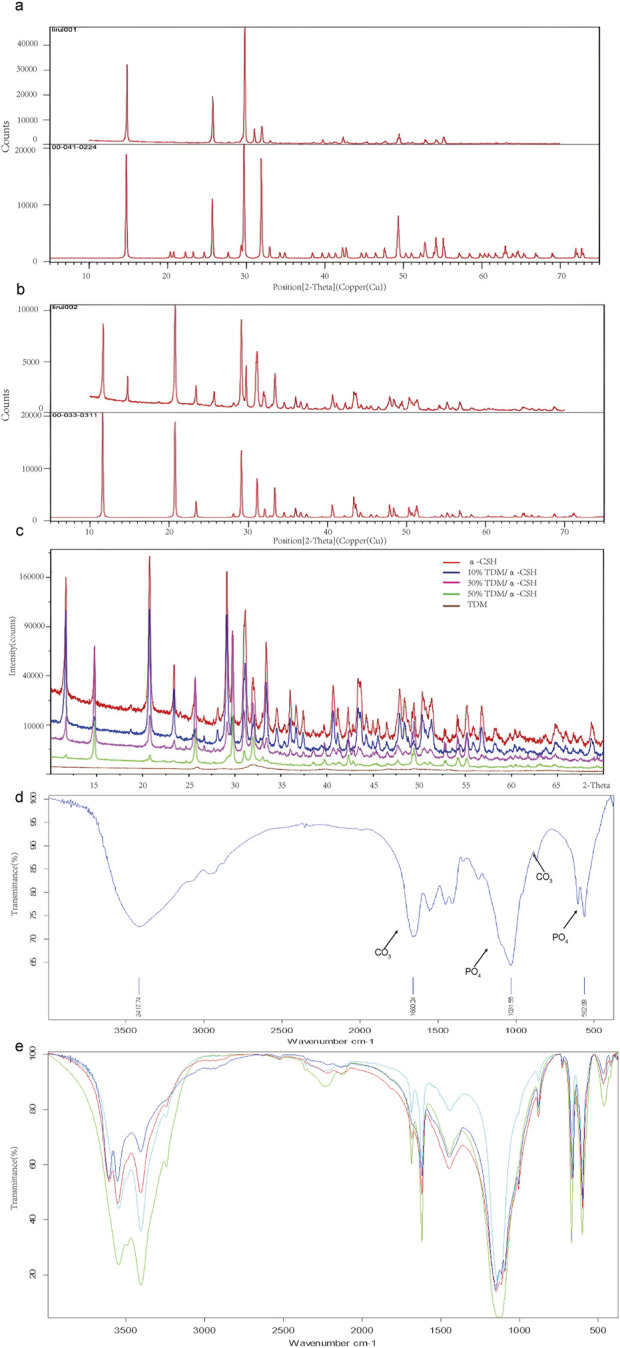
The XRD analysis of the obtained powders is presented as follows: **(A)** comparative XRD patterns of α-CSH used in this experiment (upper) versus standard α-CSH (lower). **(B)** Comparative XRD patterns of calcium sulfate dihydrate formed in this experiment (upper) versus standard calcium sulfate dihydrate (lower). **(C)** XRD patterns of α-CSH and TDM/α-CSH composites with different mass ratios (α-CSH in red, 10% TDM/α-CSH in blue, 30% TDM/α-CSH in pink, 50% TDM/α-CSH in green, and TDM in brown). Additionally, FTIR spectra for the obtained powders, including TDM **(D)**, α-CSH, and TDM/α-CSH composites with different mass ratios **(E)** are also provided.

As shown in Figure 1C, the primary application of SEM was for examination of surface particle size and crystal morphology of materials. The SEM examination images demonstrated that the method described previously was capable of adequately exposing the TDM dentinal tubules. Furthermore, the fiber bundles of the peritubular and intratubular dentin matrix loosened following treatment with EDTA (Figure 2A). The anticipated three-dimensional pore network configuration of the TDM promotes not only the even spatial distribution and proliferation of cells but also the transportation of nutrients and metabolic waste. α-CSH exhibited a unique morphology denoted by its short rod-like structure (Figure 2B). α-CSH, upon combination with the TDM, could be uniformly distributed in a biphasic structure within the cavity and surface. As the incorporation ratio of TDM increased, so did the three-dimensional porous network structure. Consequently, the combined TDM/α-CSH composite exhibited greater porosity.

The XRD patterns of α-CSH and the combined TDM/α-CSH with varying mass ratios are shown in Figures 2A–C. The characteristic diffraction peaks of α-CSH exhibited a high degree of congruence with those of the typical standard α-CSH (standard diffraction card 00-041-0224). Furthermore, with the exception of a negligible number of heteropeaks, the characteristic peaks of the new substance generated through the hydration reaction of the mixture with water aligned precisely with the standard diffraction card 00-033-0311 of calcium sulfate dihydrate. This suggests that the α-CSH utilized in our experiment was of exceptional high purity.

The diffraction outcomes of the TDM, acquired through a series of preparations, were consistent with the literature and the typical standard hydroxyapatite (Jcpds72-1243). This indicated that hydroxyapatite constituted the primary constituent of the TDM. Nevertheless, the half-peak width of the diffraction characteristic peaks was greater in the case of the TDM than that of synthetic hydroxyapatite, as indicated by the diffraction results. This discrepancy suggests that the crystallinity of the TDM was lower than that of the latter. Furthermore, the XRD spectra of the TDM/α-CSH composite, which contained varying proportions of TDM, exhibited striking resemblances to those of α-CSH alone. The characteristic peaks remained largely consistent, suggesting that the composite was formed through physical mixing and that no additional substance was produced during the mixing process.

The FTIR spectra of α-CSH, TDM, and the TDM/α-CSH composite at different mass ratios are shown in Figures 2D, E. Figure 2A confirms that hydroxyapatite is the primary component of the TDM. Furthermore, compared to synthetic hydroxyapatite, the TDM exhibited a more complex composition, containing significant amounts of CO₃, PO₄, and other organic groups. The FTIR spectra of the TDM/α-CSH composites at varying TDM ratios were remarkably similar to those of α-CSH. The characteristic peaks remained largely unchanged, indicating that the composite was formed through physical mixing, without the formation of any new substances (Figure 2B).

### 3.2 The effect of the TDM/α-CSH composite on the growth and proliferation of osteoblasts

**FIGURE 3:**
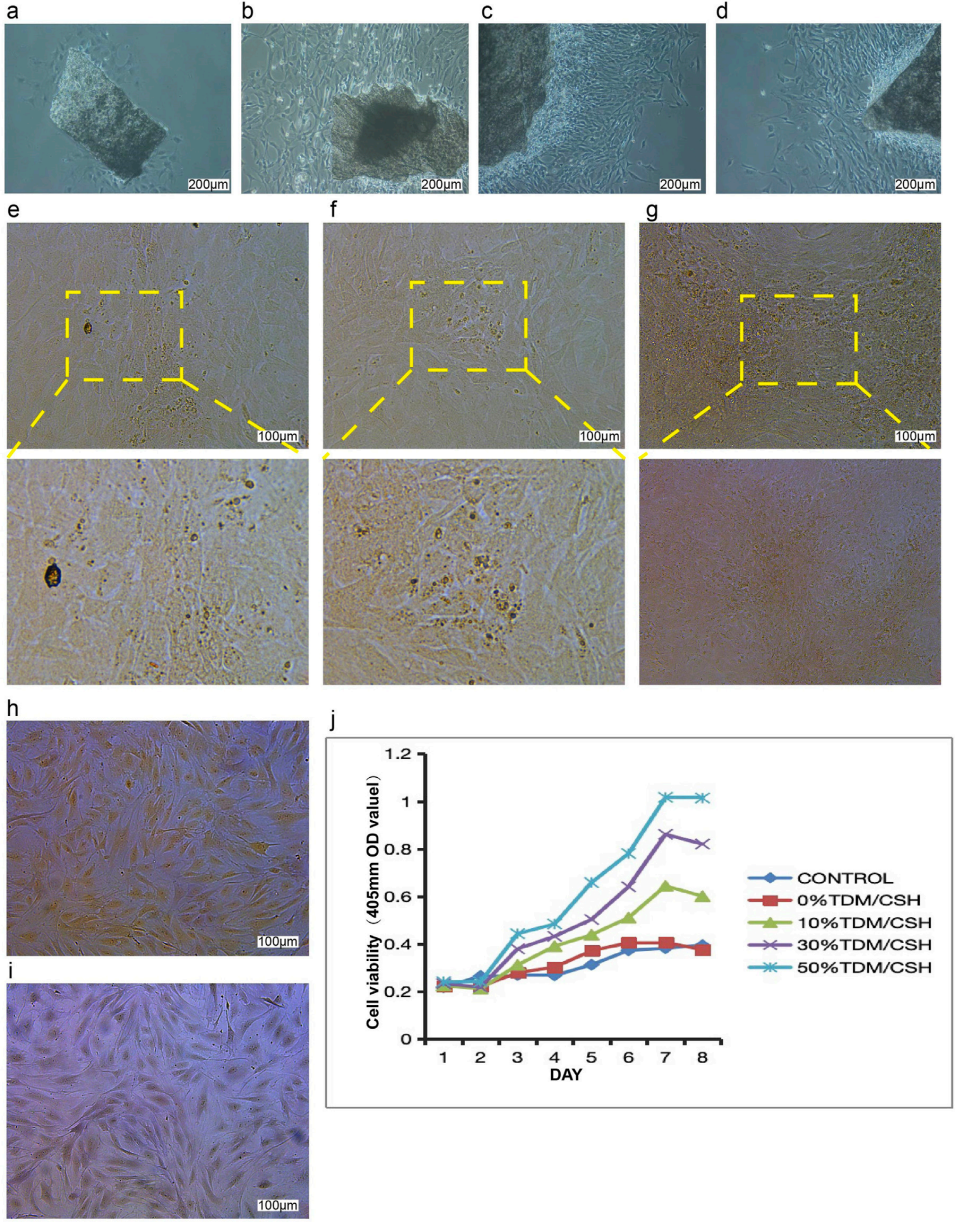
The effect of the TDM/α-CSH composite on osteoblast growth and proliferation was assessed as follows: cell morphology images of primary osteoblasts cultured for 48 h **(A)** and 7 days **(B–D)** were captured using an inverted optical microscope (100×). Mineralized nodules in osteoblasts cultured for 25 days were observed under a fluorescence microscope, with images shown in **(E–G)** after staining with Alizarin Red. Alkaline phosphatase staining images of osteoblasts cultured for 5 days are shown in **(H, I)**; **(H)** ALP staining with nuclear fixation (red staining, 100×), and **(I)** ALP staining with methyl green (100×). **(J)** Effects of material extracts on the growth and proliferation of osteoblasts.

The morphological examination of osteoblasts was done using an inverted light microscope and the image shown in Figure 3A. Following a 48 h culture period, few dispersed primary osteoblasts were seen emerging from the piece of the skull cap. The cells exhibited robust growth and seemed well-preserved. When observed under the microscope, the cells had a distinct and complete shape. They were surrounded by a transparent halo, and their elliptical cell nuclei were centrally located and surrounded by many minute, black particles. The cells had mostly irregular forms, such as shuttle, triangle, and polygon, which enabled them to establish connections with far cells via thin cell processes. As the main culture period increased, an increasing number of cells migrated out of the tissue mass and developed in a radial pattern around the bone pieces. Approximately 7–10 days later, the primary cells merged and spread throughout the plate, as shown in Figures 3B–D. In some locations, it may develop in many layers, and there was no restriction of growing contact between osteoblasts. Within 24 h of culturing, the osteoblasts securely attached to the surface of the dish and progressively increased in size. Their appearance closely resembled that of the original osteoblasts. In contrast, the dead cells were seen floating in the surrounding liquid.

Calcified nodules in osteoblasts were identified using Alizarin Red S staining. The observation of these nodules was conducted using a fluorescence microscope, as shown in Figures 3E–G. As the cells multiplied and layered on top of each other, the expanding osteoblasts merged and formed overlapping patterns, like paving stones. This resulted in the formation of many cell nodules of varying sizes. After a period of 25 days without subculture, the microscope revealed the presence of several opaque patches of varying sizes and shapes, providing evidence of mineralization in the cell nodules. Following the application of Alizarin Red S stain, mineralized sedimentation was observed in the regions where cell growth was most vigorous. These sedimentations had a mass-like appearance and radiated outward.

ALP staining was examined using a fluorescence microscope, as shown in Figures 3H, I. Under microscopic examination, dark-colored granular precipitates may be observed in the nucleus and cytoplasm of the majority of cells. The cells exhibited mostly irregular morphologies, including spindle, triangular, circular, and polygonal geometries, characterized by elongated and slender processes that facilitated connectivity with neighboring cells. Therefore, the cell morphology of the inverted microscope was confirmed once again by this staining result, which was in line with the majority of existing literature. The robust positive staining findings demonstrated a high level of purity in the isolated osteoblasts, making them suitable for following investigations.

The expansion and multiplication of osteoblasts cultivated in each batch of material extracts were evaluated using the CCK-8 assay from day 1 to day 8 (Figure 3J). The TDM/α-CSH composite material exhibited a significant increase in osteoblast proliferation compared to the empty control. This enhancement was dependent on the quantity of TDM included, and the improvement became more significant at a higher TDM concentration. Comparing the TDM/α-CSH composite extracts to α-CSH and the empty control showed that the TDM/α-CSH composite extracts significantly enhanced cell proliferation after 3 days of culture. Furthermore, as the culture duration increased, the promotion effect became more pronounced.

### 3.3 Bone regeneration of the TDM/α-CSH composite implanted in SD rats

#### 3.3.1 Observation of the gross specimen

**FIGURE 4:**
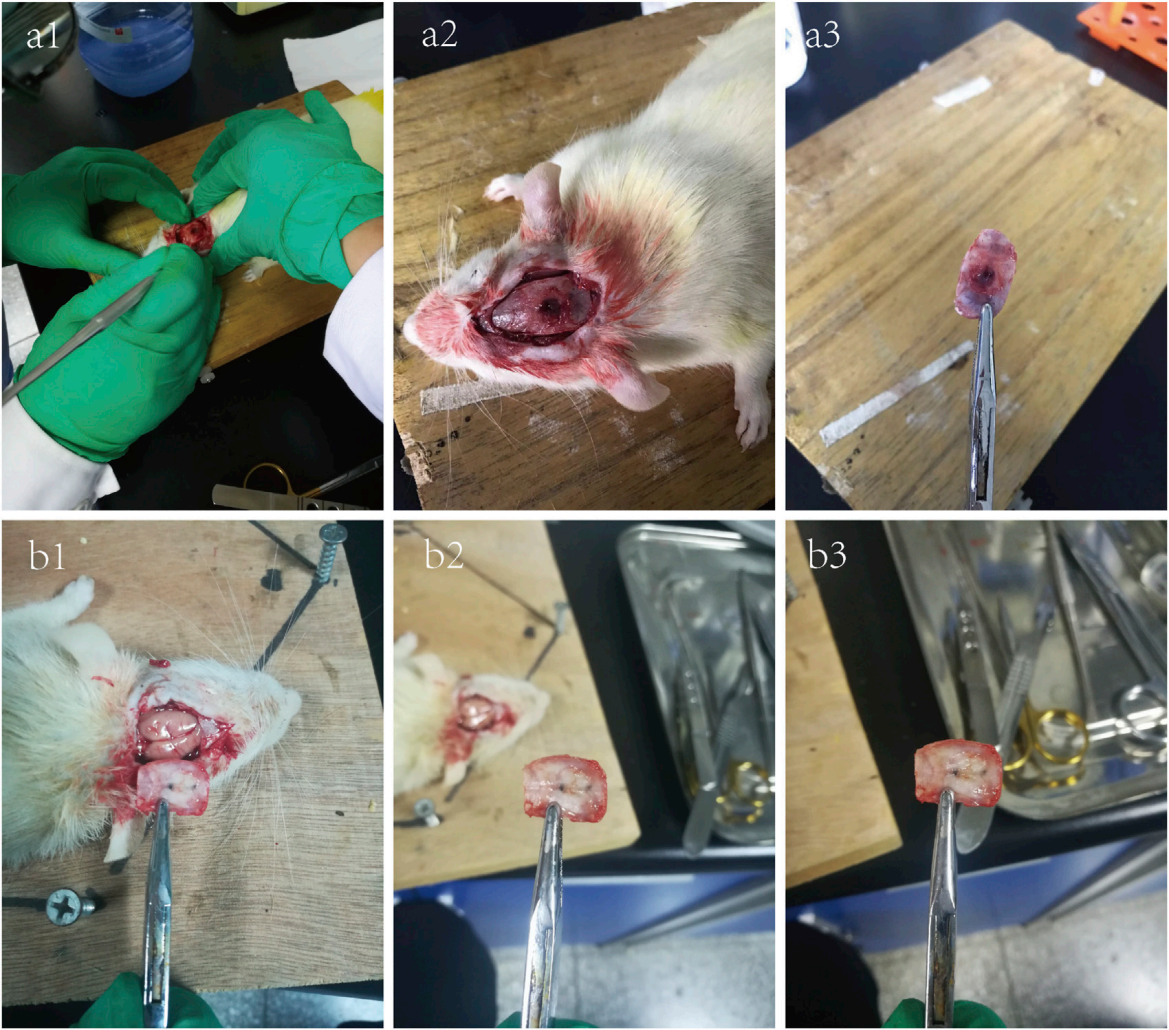
6-week **(a1–a3)** and 12-week **(b1–b3)** photographs of the critical skull defect model in SD rats.

All rats recovered well after operation, and diet and activity were normal as before. The wound healed well and was clean and dry, without signs of infection such as redness, swelling, and exudation. During the observation period, all the experimental animals were normal, and no death occurred. As shown in Figure 4 (a1–a3), at 6 weeks after operation, the bone defect had been filled with fibrous connective tissue; however, no large area of new bone calcification was formed, with no obvious inflammation found. As shown in Figure 4 (b1–b3), when the observation period was extended to 12 weeks, it was found that there was obviously more hard tissue formation in the defect area than that of 6 weeks.

#### 3.3.2 X-ray examination

**FIGURE 5:**
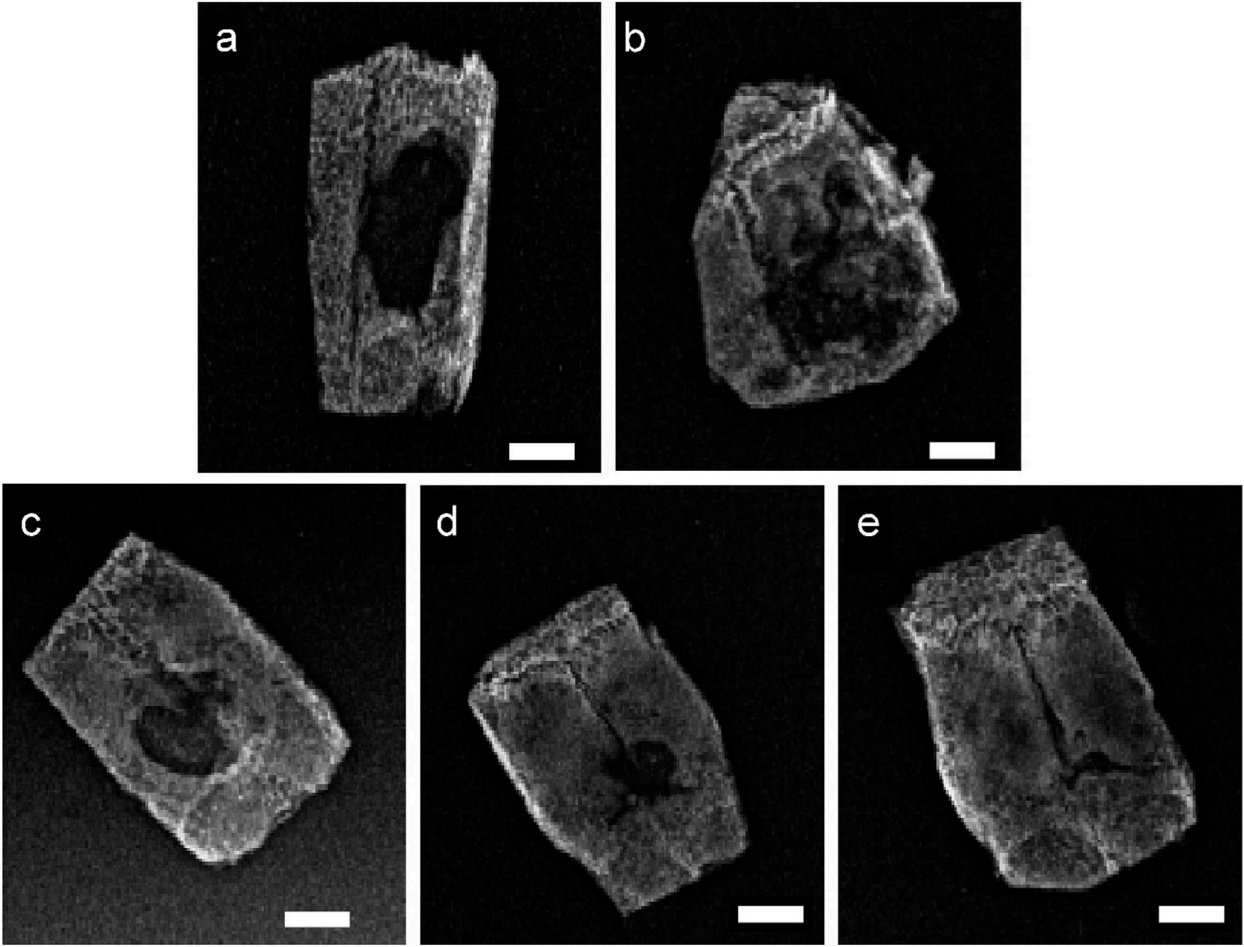
12-week X-ray photographs of the critical skull defect model in SD rats **(A)** control; **(B)** α-CSH; **(C)** 10% TDM/α-CSH; **(D)** 30% TDM/α-CSH; **(E)** 50% TDM/α-CSH). Scale bar = 5 mm.

The bone repair effect at the rat calvarial defect sites, as assessed by X-ray examination, is shown in Figure 5. In the blank control group, only minimal new bone formation was observed at the edges of the defect, while each material-treated group demonstrated higher new bone formation. The α-CSH group showed some improvement in new bone formation compared to the blank control, and the incorporation of the TDM further enhanced the composite’s ability to promote new bone formation.

#### 3.3.3 μ-CT measurement

**FIGURE 6:**
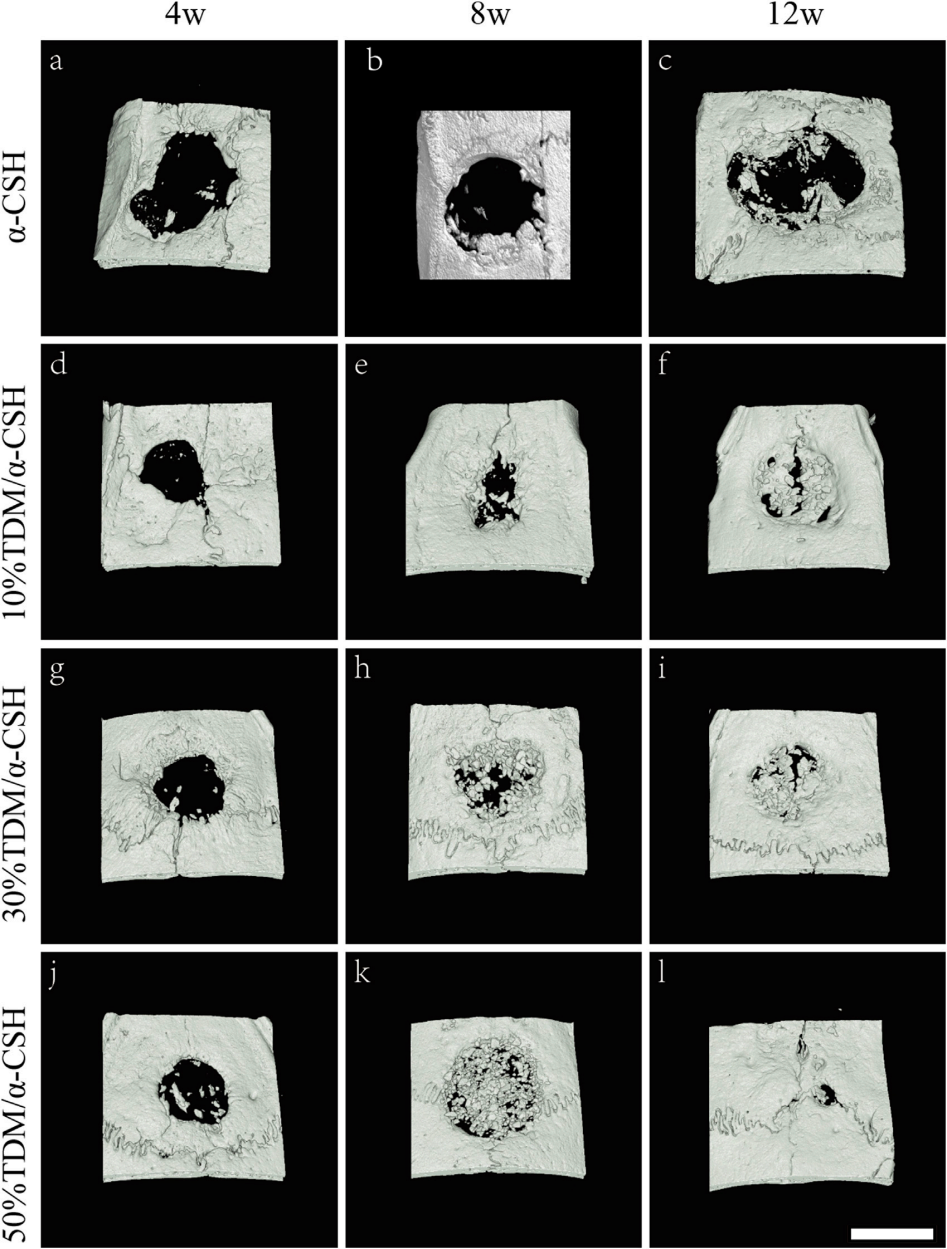
Three-dimensional reconstruction images of μ-CT scans showing critical-sized skull defects after the implantation of α-CSH **(A–C)**, 10% TDM/α-CSH **(D–F)**, 30% TDM/α-CSH **(G–I)**, and 50% TDM/α-CSH (j–l) at 4 **(A, D, G, J)**, 8 **(B, E, H, K)**, and 12 **(C, F, I, L)** weeks post-surgery. Scale bar = 5 mm.

To confirm the implanted material resorption and new bone formation in the calvarial defects of rats, μ-CT was performed, and 3D images were generated to observe the morphology of the regions of interest and evaluate the reconstruction of the newly formed bone for different periods of time. As shown in Figure 6, it could be observed that all the bone defects in different groups were gradually repaired over time. Four weeks after implantation, new bone formation in all defects was observed to be relatively modest, although some improvement was noted. By 8 weeks post-implantation, the TDM/α-CSH groups demonstrated a more pronounced area of new bone formation than the α-CSH group. Notably, 12 weeks after surgery, the 50% TDM/α-CSH group showed a significant increase in new bone formation, creating a marked contrast with the other groups.

#### 3.3.4 Immunohistochemical analysis

**FIGURE 7:**
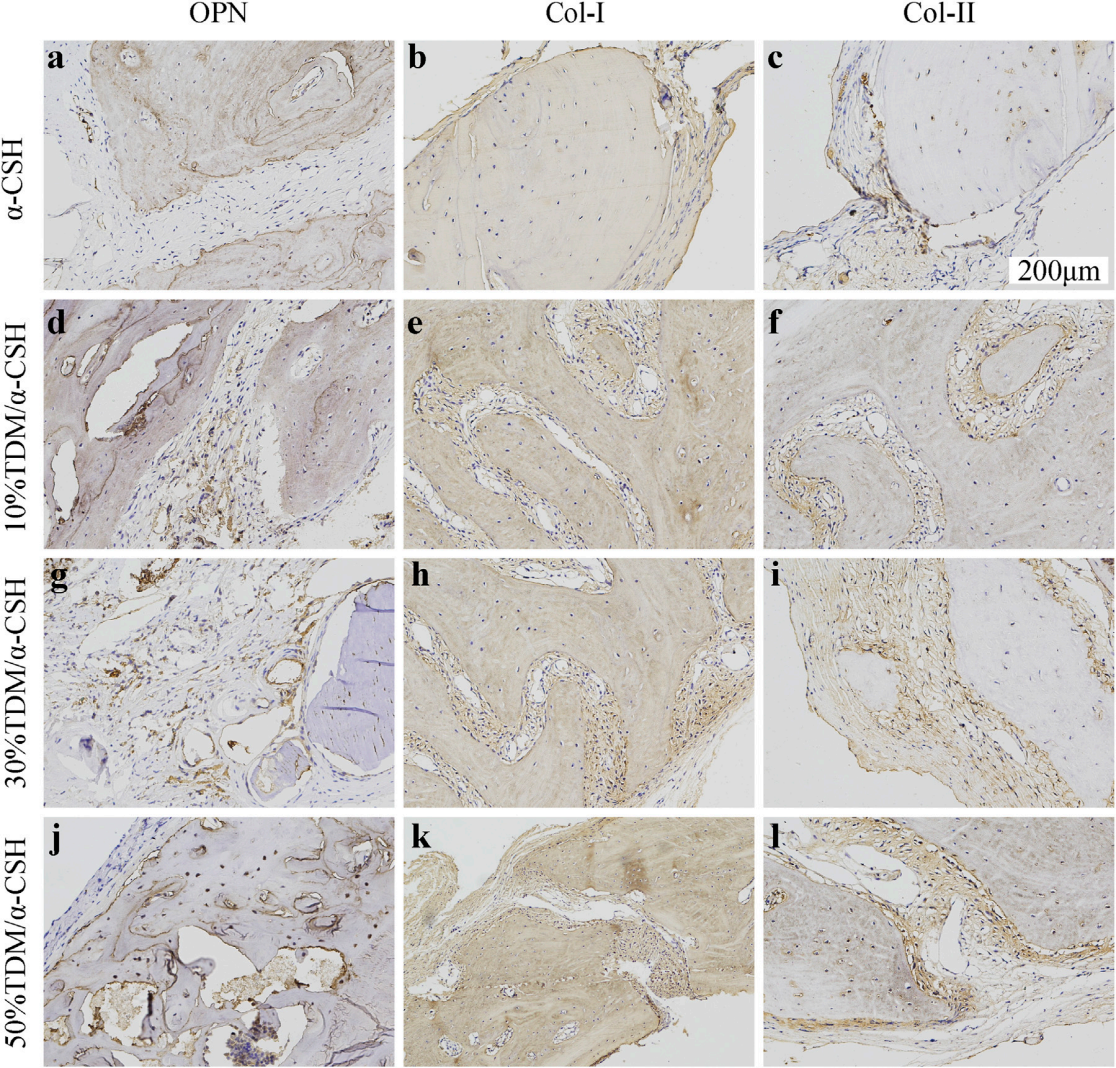
Immunohistochemical analysis of OPN **(A, D, G, J)**; (200×), COL-I **(B, E, H, K)**; (200×), and COL-II **(C, F, I, L)**; (200×) in α-CSH, 10% TDM/α-CSH, 30% TDM/α-CSH, and 50% TDM/α-CSH implanted in critical skull defects at 12 weeks. Yellowish brown staining and red arrows indicate positive expression, while blue arrows indicate residual material. Scale bar = 200 μm.

The immunohistochemical analysis was conducted to confirm new bone formation *in vivo*, using positive expressions of osteoblastic markers such as Col-I, collagen type II (Col-II), and OPN across different groups and time periods. As shown in Figure 7, minimal OPN expression was observed in the α-CSH group. In contrast, the TDM/α-CSH groups displayed significantly higher positive expression, particularly at the edges of the fracture gaps, material fragments, and near the cement interfaces. The intensity of OPN staining increased proportionally with the TDM content. Similarly, stronger staining of Col-I and Col-II was observed in the TDM/α-CSH groups within the calvarial defect areas, in stark contrast to the α-CSH group. Furthermore, the immunohistochemical analysis indicated that positive staining intensity in all groups increased over time post-implantation. These findings indicate that increasing the TDM content in the composite material promotes higher expression of osteoblastic markers, such as Col-I, Col-II, and OPN, thereby promoting new bone formation.

## 4 Discussion

The composition of dentin is very similar to that of normal bone tissue, and their biological immunogenicity is extremely low because they do not contain cell bodies, thus exhibiting good biosafety (Kim et al., 2013). Meanwhile, there are many bioactive proteins and factors related to osteogenesis and odontogenesis in the dentin matrix, and implanting these in the bone tissue defect area can provide a microenvironment for the induction of new bone formation (Li et al., 2011; Bhatt and Rozental, 2012). Extensive studies have demonstrated that partially demineralized dentin exhibits superior osteogenic capacity and osteoconductivity, even surpassing those of HA (Dioguardi et al., 2015; Kim et al., 2007). Our study’s TDM, obtained through a gradient demineralization method, conforms to established standards, such as Jcpds72-1243. Our preliminary tests confirmed the presence of osteogenic factors like BMP-2, DMP1, osterix, and Cbfal (Runx2) in the TDM. Through proteomic and mass spectrometry analyses, we further verified that these factors could be secreted into the surrounding environment, stimulating stem cell growth and osteogenic differentiation (Li et al., 2011; Yang et al., 2017b).

α-CSH has been widely utilized in clinical settings due to its low cost, structural uniformity, *in situ* solidification, and ease of molding. It also demonstrates excellent osteoconductivity, biocompatibility, and biodegradability (Turner et al., 2003; Strocchi et al., 2002; Kim et al., 2011; Thomas and Puleo, 2009). Commercial products, such as Surgiplaster, OsteoSet, and Stimulan, are commonly used as calcium sulfate-based bone graft substitutes (Saul, 1966; Sidqui et al., 1995). In our study, we combined the TDM and α-CSH at mass ratios of 10%, 30%, and 50%, hypothesizing that this composite could enhance bone formation in critical-sized rat calvarial defects.

Mechanical strength is essential for bone substitute materials in the repair of bone defects (Cavelier et al., 2020). This study showed that both the average compressive strength and elastic modulus of TDM/α-CSH cements, measured using the universal mechanical testing machine, decreased with increasing TDM content compared to pure α-CSH. The primary source of mechanical strength in the composite comes from the calcium sulfate dihydrate formed during the hydration of α-CSH, which creates a compact solid with high compressive strength (Salvatore et al., 2012; Nilsson et al., 2003). The TDM, unable to solidify independently, reduces the crystal crosslinking rate of α-CSH. Furthermore, the loose and porous surface of the TDM contributes to voids in the solidified composite, decreasing its mechanical strength. Optimizing the TDM ratio to balance mechanical properties and osteogenic potential would enhance the composite’s clinical utility.

SEM examination revealed that the TDM dentinal tubules were sufficiently exposed, and the fibers in the peritubular and intratubular dentin matrix loosened after EDTA treatment. The α-CSH infiltrated the porous TDM structure, creating a biphasic composite. As the TDM ratio increased, the composite exhibited greater porosity, improving surface roughness and enhancing cell adhesion, growth, and spreading. This 3D porous network is ideal for bone defect repair, facilitating new bone formation, nutrient flow, and waste removal. However, excessive porosity compromises compressive strength, while insufficient porosity hinders bone and vascular ingrowth (Cao et al., 2017; Mao et al., 2012; Bohner, 2010; Heini and Berlemann, 2001; Zheng and Liu, 2005; Cornell and Lane, 1998; Katoh et al., 1993). The XRD and FTIR analyses revealed that the composite is a physical mixture, retaining the original properties of the α-CSH and TDM without forming new compounds. The TDM primarily consists of hydroxyapatite but also contains organic groups like CO_3_ and PO_4_, distinguishing it from synthetic HA.

Our study also showed that TDM/α-CSH composites exhibited no cytotoxicity and enhanced osteoblast proliferation, with the effect increasing as the TDM content increased. This is likely due to the release of active proteins and factors from the TDM. Mass spectrometry and proteomic analyses confirmed the continuous release of bioactive factors, such as Col-I, BMP-2, DMP1, and TGF-β1, which are essential for osteogenesis (Li et al., 2011; Yang et al., 2017b). ELISA results further indicated that the TDM released VEGF, BMP-2, TGF-β, and PDGF at a steady rate, which promoted the expression of Col-I and Runx2 in BMSCs, enhancing osteogenesis (Li et al., 2011; Chen et al., 2015; Guo et al., 2012).

In order to explore the clinical application potential of the TDM/α-CSH composite cement, an *in vivo* animal study in critical calvarial bone defects of SD rats was performed. Gross observation is shown in Figure 4. When the observation period was extended to 12 weeks, increased hard tissue formation and calcification were observed in the defect area than that of 6 weeks filled with fibrous connective tissue. *In vivo* studies in SD rats demonstrated that higher TDM content in the composite significantly enhanced new bone formation. X-ray and μ-CT results, along with immunohistochemical analysis, indicated increased osteoblast marker expression with higher TDM content, highlighting its potential for clinical application. Additionally, immunohistochemical analysis of histological sections shown in Figure 7 revealed that higher TDM content in the composite material led to the increased expression of osteoblastic markers such as Col-I, Col-II, and OPN, which contributed to higher new bone formation. In recent years, some inorganic elements, growth factors, bone marrow aspirates, bioactive proteins, and some stem cells with osteogenic differentiation direction have been recombined with artificial bone transplantation materials, providing them with certain osteoinductive properties after being implanted into bone defects, which is one of the hotspots that many scholars and researchers pay attention to at present (Vo et al., 2012; Mehta et al., 2012; Bansal et al., 2009). After the TDM blended into α-CSH, it is equivalent to loading the biological information of osteogenesis on the composite material, which can provide a certain osteogenic microenvironment when implanted into the bone defect area, resulting from the release of the aforementioned osteogenic-related active proteins and factors by the EDTA-treated dentin matrix. The literatures show that α-CSH has a relatively rapid degradation rate, and the pore structure as a good bone conduction stent of composite cement will be exposed with the continuous absorption and degradation of α-CSH, which not only contributes to the transport of nutrients and metabolic wastes but also provides an excellent scaffold space for cell adhesion, growth, new bone formation, and vascular growth in the defect area (Nilsson et al., 2003; Cao et al., 2017). In addition, some studies have shown that the osteogenesis of α-CSH may be related to the decrease in the local pH value and the formation of a high-calcium microenvironment during degradation. The acidic environment causes the local decalcification of bone tissue around the receptor defect area, which leads to the release of related osteogenesis active proteins and factors contained in the bone matrix. Furthermore, the high-calcium microenvironment can not only provide a sufficient calcium source for new bone formation but also promote the growth and proliferation of osteoblasts (Yu et al., 2009). Turner et al. (2003) confirmed that a high-calcium microenvironment can inhibit the proliferation and differentiation of spleen-derived osteoclasts.

Although our findings confirm the effectiveness of TDM/α-CSH composites in bone defect repair, further research is needed to optimize the TDM ratio and elucidate the molecular mechanisms underlying its osteogenic potential. The large gradient in the TDM content used in this study raises questions about whether more TDM consistently enhances bone formation or whether an optimal ratio exists for clinical efficacy.

## 5 Conclusion

In this study, we synthesized TDM/α-CSH composite cement for treating critical-sized calvarial bone lesions in rats. The average compressive strength and elastic modulus of the TDM/α-CSH cements, as measured using universal mechanical testing equipment, decreased as the TDM content increased, compared to pure α-CSH. The TDM/α-CSH composite exhibited a three-dimensional porous network structure with enhanced surface roughness. XRD and FTIR analyses confirmed that the combination of the two materials resulted in a physical mixture without the formation of new substances during blending. Notably, the TDM/α-CSH composites demonstrated excellent biocompatibility and a remarkable capacity to promote osteoblast growth, which was proportional to the TDM content. In an *in vivo* calvarial bone defect model, TDM/α-CSH cements significantly enhanced new bone formation, as verified by X-ray and μ-CT analyses. These findings strongly suggest that incorporating the TDM into α-CSH improves the bone regeneration properties of the composite, making TDM/α-CSH cements a promising option for bone defect repair.

## Data Availability

The original contributions presented in the study are included in the article/Supplementary Material; further inquiries can be directed to the corresponding author.
